# Harnessing the evolutionary information on oxygen binding proteins through Support Vector Machines based modules

**DOI:** 10.1186/s13104-018-3383-9

**Published:** 2018-05-11

**Authors:** Selvaraj Muthukrishnan, Munish Puri

**Affiliations:** 1 Institute of Microbial Technology (CSIR-IMTECH), Sector-39A, Chandigarh, 160036 India; 2 Protein Biotechnology Laboratory, Department of Biotechnology, Punjabi University, Patiala, India; 3 Present Address: Centre for Marine Bioproducts Development, College of Medicine & Public Health, Flinders University, 4.23/Level 4 Health Sciences Building, Registry Road, Bedford Park, Adelaide, 5042 Australia

**Keywords:** Oxygen binding proteins, Hemoglobin, Myoglobin, Leghemoglobin, Erythrocruorin, Hemerythrin, Hemocyanin, Support Vector Machines, Confusion matrix, ROC Analysis

## Abstract

**Objectives:**

The arrival of free oxygen on the globe, aerobic life is becoming possible. However, it has become very clear that the oxygen binding proteins are widespread in the biosphere and are found in all groups of organisms, including prokaryotes, eukaryotes as well as in fungi, plants, and animals. The exponential growth and availability of fresh annotated protein sequences in the databases motivated us to develop an improved version of “Oxypred” for identifying oxygen-binding proteins.

**Results:**

In this study, we have proposed a method for identifying oxy-proteins with two different sequence similarity cutoffs 50 and 90%. A different amino acid composition based Support Vector Machines models was developed, including the evolutionary profiles in the form position-specific scoring matrix (PSSM). The fivefold cross-validation techniques were applied to evaluate the prediction performance. Also, we compared with existing methods, which shows nearly 97% recognition, but, our newly developed models were able to recognize almost 99.99 and 100% in both oxy-50 and 90% similarity models respectively. Our result shows that our approaches are faster and achieve a better prediction performance over the existing methods. The web-server Oxypred2 was developed for an alternative method for identifying oxy-proteins with more additional modules including PSSM, available at http://bioinfo.imtech.res.in/servers/muthu/oxypred2/home.html.

**Electronic supplementary material:**

The online version of this article (10.1186/s13104-018-3383-9) contains supplementary material, which is available to authorized users.

## Introduction

Oxygen is an essential part of the atmosphere and is necessary to sustain the most terrestrial life of living organisms as it used in respiration and regulation of a variety of cellular functions. The oxygen binding proteins (oxy-proteins) of various organisms considerably differ from one another and classified mainly on their structure and physiochemical properties as hemoglobin, hemocyanin, hemerythrin, myoglobin, leghemoglobin, and erythrocruorin. Each oxy-proteins have its own functional characteristics and structure with unique oxygen-binding capacity [1–11].

A number of computational methods have been proposed for identifying functional proteins on their primary sequences using machine learning approaches [12–14]. These methods are always needful to improve or to find new features for identifying protein family and their classes, sub-classes to avoid negative prediction or to reduce false positive rates.

In 2007, Muthukrishnan et al. developed Oxypred method for predicting oxygen-binding proteins using the simple amino (AC) and dipeptide composition (DC). The growing of protein sequence databases and availability of newly annotated sequences of oxy-proteins in the post genomic era, retrospectively encouraged us to introduce a new improved version of forged oxypred method. An attempt was made to include a recently generated highly non-redundant dataset in the development of Oxypred2 with a different protein features [15]. Recently, it has observed that the use of evolutionary profile in the form of a position-specific scoring matrix (PSSM) predicted various functional proteins with a higher accuracy [16, 17]. Hence, we applied many approaches, including the PSSM based evolutionary profile to improve prediction quality of oxy-proteins.

In this study, recently generated two different cut-off non-redundant datasets 50 and 90% were applied to develop Oxypred2. The difference between current and previous study reflected that PSSM and Hybrid approach, confusion matrix analysis, prediction score graphs, and ROC analysis has been added as extra features.

The many different prediction features are always important to understand their functional behavior aspects [18–21]. Here, we compared prediction performance of 50 and 90% similarity datasets in all modules to find the best identification of oxy-proteins. The prediction results and their complete analysis show that the developed method Oxypred2 is an improved version and alternative method for identifying oxy-proteins.

## Main text

### Methods

#### Datasets

The two different datasets sequences (90 and 50%) were extracted from UniProt databases by searching the individual keyword of oxy-proteins [22]. The final dataset contains 2498 and 5474 sequences as in 50 and 90% respectively. In sub-class, 47–114 erythrocruorin, 42–154 hemocyanin, 1378–2585 hemerythrin, 957–2462 hemoglobin, 34–34 leghemoglobin and 40–125 myoglobin as in both 50 and 90% datasets respectively. Due to less availability, 90% leghemoglobin dataset used for 50% dataset. The independent non-oxy protein datasets were constructed according to the size of oxy-proteins by selecting randomly as 2565 and 5499 on 50 and 90% cutoff datasets respectively.

#### Support Vector Machines

In this study, free downloadable package of SVM-light was used to generate modules [23, 24]. It has been successfully applied to numerous classification and pattern recognition problems such as classification of protein secondary structure, subcellular localization, DNA-binding, ATP-binding and transporter family protein predictions [25–33].

#### PSSM-profile

The PSSM profile provides the evolutionary information about residues conservation at a given position in a protein sequence. The construction of PSSM profile was generated using GPSR package available at http://www.imtech.res.in/raghava/gpsr/. We applied GPSR programs for PSI-BLAST searches against the non-redundant (nr) database using different iterations with a cutoff *E* value 0.001 [34, 35]. Further, each value has been normalized the range between 0 and 1 by the following equation,

1 $$Normalized\,value = \frac{{\left( {Value - Minimum} \right)}}{{\left( {Maximum - Minimum} \right)}}$$

In 0–1 value, minimum scores consider as “0,” and the maximum scores become “1”.

#### Evaluation models

We applied fivefold cross-validation techniques, as it was done by many investigators with SVM as the prediction engine. In this technique, the dataset was divided into five sets consisting of nearly equal number of sequences, where four sets used for training and remaining set for testing. The training and testing set was carried out five times in such a way that each part was used once for testing, and the whole process was repeated 20 times.

The objectives of our classifieds are to discriminate the oxy-protein from those of negative discipline, and the following terminology used to evaluate of our classifier as,

- True positive (TP)—a protein is identified as an oxy-protein by both classifier and oxy-proteins model.
- True negative (TN)—a protein is not identified as a oxy-protein by either the classifier or oxy-protein model.
- False positive (FP)—a protein is identified as positively as oxy-protein by the classifier, but not by the oxy-protein model.
- False negative (FN)—a protein is identified as oxy-protein by the oxy-protein model but not by the classifier.

In order to assess the prediction performances, accuracy (ACC), Mathew’s correlation coefficient (MCC), sensitivity (Sen) and specificity (Sep) were calculated using standard Eqs. (2–5) [36–38],

2 $$Accuracy\,\left( {ACC} \right) = \frac{TP + TN}{TP + TN + FP + FN}$$

3 $$Sensitivity\,\left( {SN} \right) = \frac{TP}{TP + FN}$$

4 $$Specificity\,\left( {SP} \right) = \frac{TN}{TN + FP}$$

5 $$MCC = \frac{TP \times TN - FP \times FN}{{\sqrt {\left( {TP + FP} \right)\left( {TP + FN} \right)\left( {TN + FP} \right)\left( {TN + FN} \right)} }}$$

### Results

**Fig. 1:**
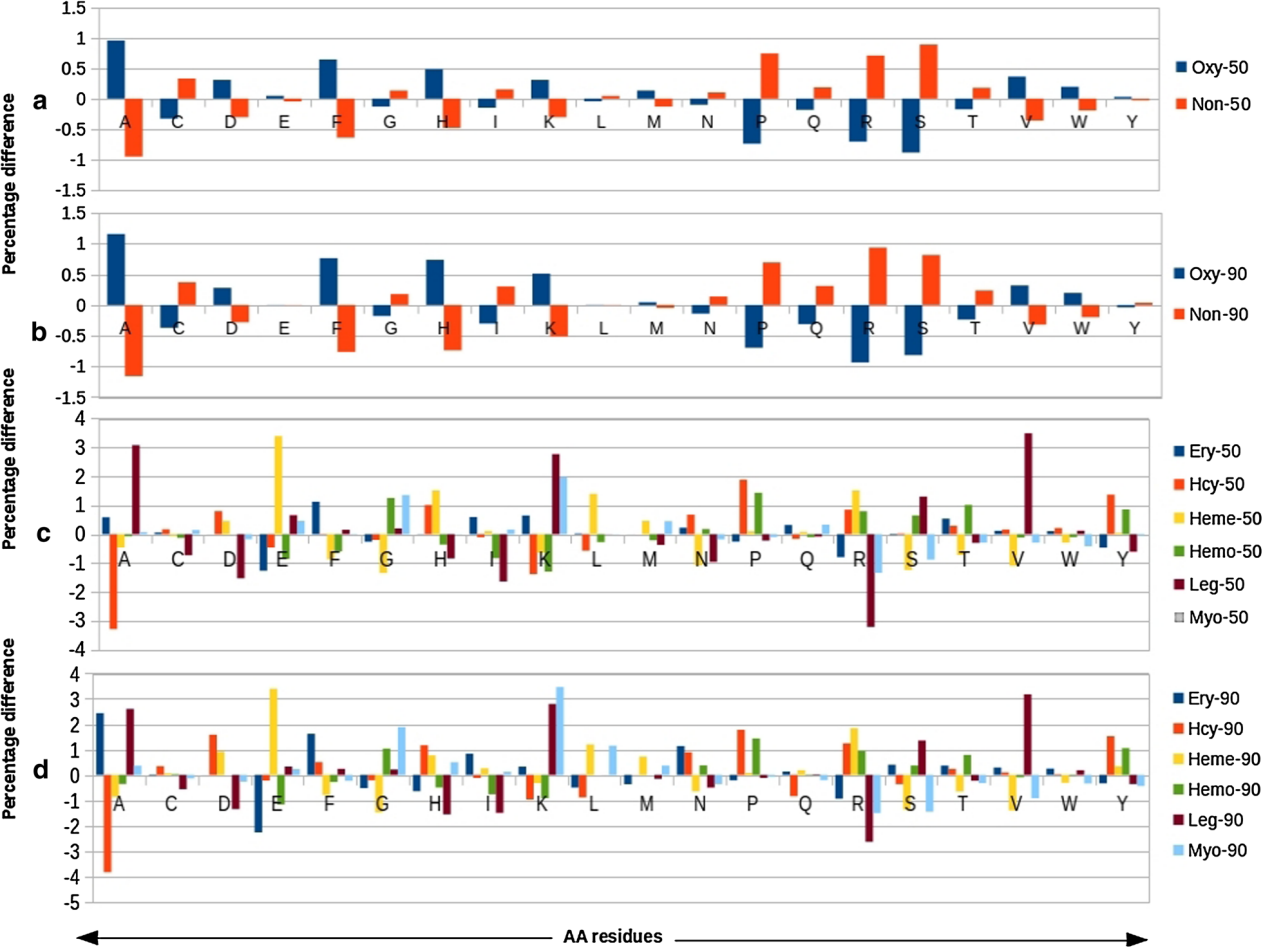
Amino acid distribution difference between oxy and non-oxy sequences: It has been calculated based on median scores. **a** Difference between oxy-50 and non-50. **b** Difference between oxy-90 and non-90. **c** Differences within the oxy-sub-classes of oxy-50 datasets. **d** Differences within the oxy-sub-classes of oxy-90 datasets

Determining the relative amino acid composition will give a characteristic profile for protein [39]. Here, we calculated average AC composition of oxy-proteins according to their median scores. We observed that the residues Ala and Phe are present > 0.5% in oxy-50 sequences, which compared to non-oxy-50% sequences. In oxy-90 residues Ala, Phe, His and Lys are more 0.5% than non-90 sequences. In the oxy-50 classification dataset, residues Ala, Lys and Val are > 2, 3, 2% in Leg, hemo, and myo. Ala and Arg residues are very less (− 3%) in Hcy-50 and Leg-50 sequences respectively. In 90% oxy-datasets, Ala residue is 2% more in Ery-90 and leg-90, Glu, Lys, and Val are present 3% more in heme, myo, and leg proteins respectively. Ala, Glu, and Arg are less 2% in hcy, ery and leg proteins, results shown in Fig. 1, Additional file 1: Figure S1 and Additional file 2: Figure S2. In sub-classes, sequence length profile of oxy-50 and 90 were compared, found most of the sequences of heme and hemo proteins belong to the range between 101 and 200. The other proteins are distributed in different length ranges (Additional file 3: Figure S3).

In AC approach prediction, we achieved the maximum accuracy was 82.05, and 87.79% in oxy-50 and oxy-90 datasets. DC-method, maximum accuracy was 80.42 and 84.81% in oxy-50 and oxy-90 respectively. The complete prediction results are shown in Additional file 4: Table S1, and the classification approach results shown in Table 1. The evolutionary profile based PSSM method have been applied to many functional protein predictions [40, 41]. In PSSM methods achieved the maximum accuracy was 85.10 and 81.81% in oxy-50 and oxy-90 datasets respectively. We observed that, in classification the PSSM method prediction accuracy was slightly increased in Ery, Hcy, Heme, Leg, and Myo in oxy-50 than the oxy-90 datasets.

**Table 1:** The performance of oxy-proteins sub-class SVM-models (Ery, Hcy, Heme, Hemo, Leg and Myo) in different approach and comparison between oxy-50 and oxy-90 output data

	ACC		Sen		Sep		MCC	
	50%	90%	50%	90%	50%	90%	50%	90%
Ery								
AC	95.65	97.14	34.03	67.33	96.79	97.75	0.53	0.80
DC	90.29	93.26	55.56	94.32	90.93	93.24	0.65	0.93
PSSM	94.15	93.56	64.58	90.91	94.69	93.61	0.76	0.92
AC–DC	90.43	89.17	61.11	94.03	90.97	89.07	0.70	0.91
Hcy								
AC	97.18	98.06	100.00	92.92	97.14	98.20	0.99	0.95
DC	93.36	95.09	98.44	94.17	93.28	95.12	0.96	0.94
PSSM	94.40	90.41	100.00	95.63	94.31	90.27	0.97	0.92
AC–DC	93.19	94.02	100.00	94.38	93.08	94.01	0.97	0.94
Heme								
AC	86.25	91.73	92.57	94.90	78.49	88.89	0.79	0.90
DC	89.57	93.21	98.98	99.26	78.01	87.79	0.87	0.93
PSSM	90.09	89.00	99.07	99.55	79.07	79.56	0.88	0.89
AC–DC	87.41	90.55	98.82	99.31	73.41	82.69	0.84	0.90
Hemo								
AC	82.99	87.43	87.80	94.87	80.01	81.34	0.77	0.85
DC	84.95	89.85	93.65	98.64	79.55	82.67	0.89	0.89
PSSM	87.26	88.17	97.78	99.24	80.74	79.12	0.88	0.88
AC–DC	83.49	87.08	96.92	99.09	75.16	77.26	0.84	0.87
Leg								
AC	98.76	99.13	97.92	100.00	98.77	99.12	0.99	0.99
DC	94.50	93.78	100.00	100.00	94.44	93.75	0.97	0.97
PSSM	98.25	97.43	100.00	100.00	98.23	97.42	0.99	0.99
AC–DC	96.84	94.53	100	100.00	96.81	94.50	0.98	0.97
Myo								
AC	92.92	96.62	59.38	86.50	93.47	96.86	0.71	0.91
DC	89.60	92.19	62.50	92.25	90.05	92.19	0.70	0.92
PSSM	93.06	91.02	76.56	90.75	93.33	91.03	0.83	0.90
AC–DC	86.60	85.95	67.19	91.50	86.91	85.82	0.71	0.87

*AC* Amino acid composition, *DC* dipeptide composition, *PSSM* position specific scoring matrix, *AC–DC* hybrid profile, *ACC* accuracy, *Sen* sensitivity, *Sep* specificity, *MCC* Matthews correlation coefficient

Further, to improve the prediction accuracy, a Hybrid approach based modules were developed [42]. The prediction accuracy was 81.73 and 83.51% in oxy-50 and oxy-90 respectively. In classification, Hcy, Heme, Hemo accuracy were slightly increased in oxy-90 than oxy-50. Overall, DC and Hybrid method prediction results are shows similar in oxy-50 and oxy-90, and it doesn’t show any significance differences (Table 1).

**Fig. 2:**
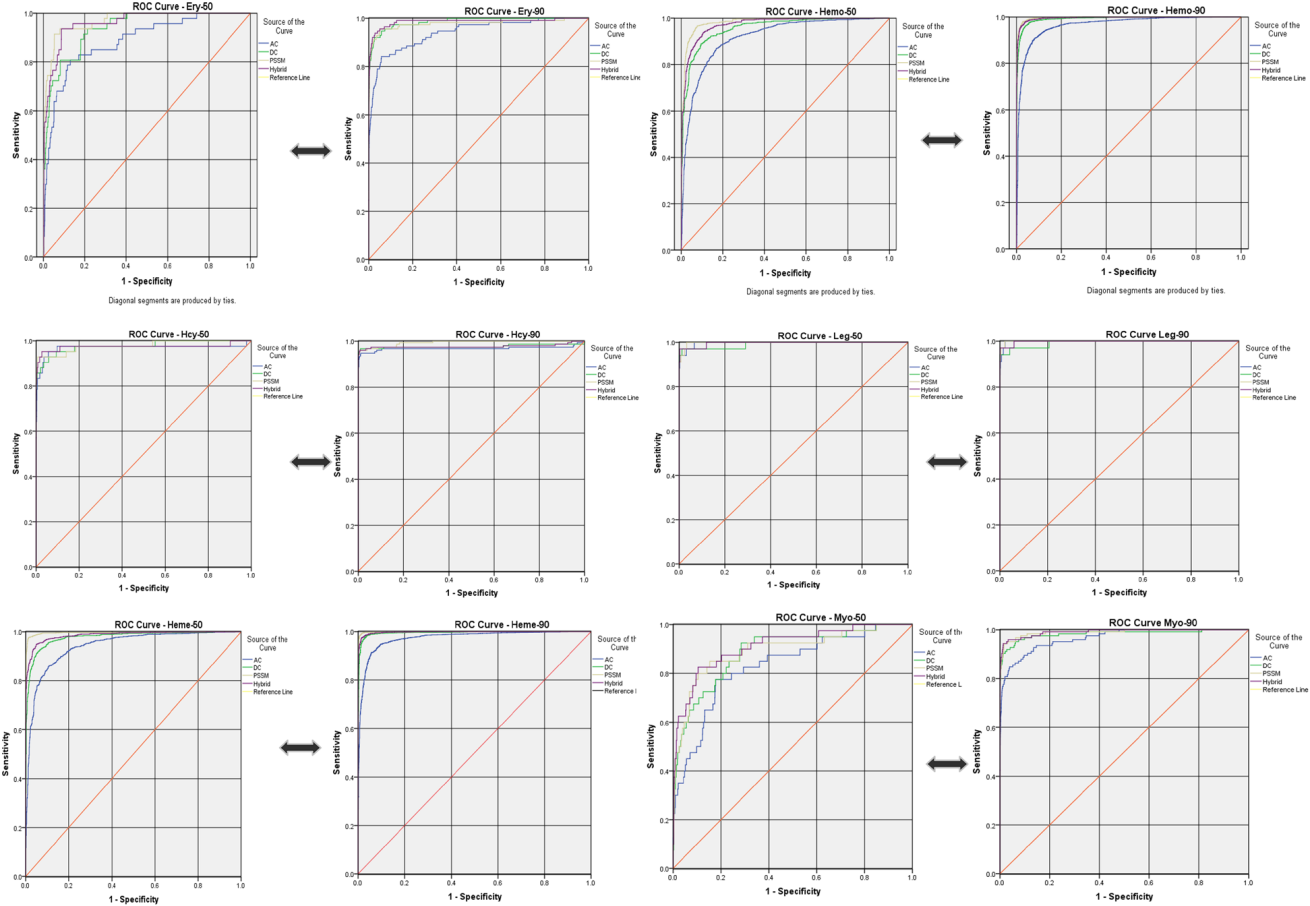
ROC curve oxy-classification in all approaches. The performance of oxypred2 developed models by ROC plots in all oxy sub-classes. The area under curve was measured for all approached models

In order to verify the prediction performance of their developed models, we also did the ROC analysis with our original data, and achieved area under the curve (AUC) 0.894 and 0.959 in oxy-50 and oxy-90 (Additional file 5: Figure S4), in classification AUC’s shown in Additional file 4: Table S2 and Fig. 2. In addition, a confusion matrix based prediction scores graphs were generated [43], to cross-check the developed model’s performance on original data. According to our results, no miss-classifications occurred in the proposed models; it means no positive sequence identified as negative and no negative sequence defined as positive. So that, our developed models are good in recognizing the positive and negative sequences.

At the same time, classification based models also doing the best performance recognizing positive and negative sequences. Eventhough, some sequence couldn’t identified by their own class models, rather identified by other class models. In oxy-50 datasets 3-Ery, 10-Hemo and 5-Myo sequences are not recognized by their models in all approaches. Rather, it recognized by other sub-class models. In oxy-90 datasets, 2, 4, 2 sequences of Ery, Hemo and Myo are confused and not recognized by their models, but identified by other models. Interestingly, some sequences of Ery, Hemo, and Myo are not identified by their models and other models too. The complete confusion matrix results of both oxy-50 and oxy-90 shown in Additional file 4: Table S3. The prediction score graphs are mainly developed to show the performance of models in separation of positive and negative sequences. According to the graphs, separation with maximum margins shown in DC, PSSM and Hybrid approaches. However, the confusion matrix result shows that some sequences are very similar between Ery, Hemo, Myo, and these sequences may be evolutionary important (Additional file 6: Figure S5 and Additional file 7: Figure S6).

Also, we compared prediction profile performance of accuracy, sensitivity, and specificity on threshold level. We found that most of the classes are showing better performance in the 0–1.5 thresholds, mostly the ACC, Sen and Sep scores are associated with a particular point threshold, but few of them doesn’t show any connections over the thresholds. Ery-50 and 90 AC data’s are not showing association with ACC, Sen, and Sep, but in DC and PSSM approaches, both Ery-50 and Ery-90 data’s are having connections in negative thresholds. Interestingly, in hybrid approach, Ery-50 data shown in negative threshold, but Ery-90 appeared at positive threshold. In Hcy Class, AC-90 data shown at negative threshold, rest all approaches appears in positive threshold (0–1.5). However, all Heme and Hemo class data’s are joining in positive threshold in all approaches. In Leg class, only AC-50 shown in negative and rest all approaches in positive threshold. In Myo class, AC-50 does not shown cross, but DC-90 and PSSM-50 at “0” threshold. Hybrid-90 shown in positive threshold and all other approaches in negative thresholds. Moreover, in most cases, accuracy and specificity data’s are similar (Additional file 8: Figure S7).

In Oxypred2 study, average ACC, Sen, and Sep from − 1.5 to +1.5 thresholds and compared the performance of both oxy-50 and oxy-90 sub-classes in all approaches. We observed that, Ery and Myo sensitivity data increased in oxy-90 than oxy-50. Moreover, all sub-classes showing more than 80% ACC, Sen and Sep in oxy-90. In oxy-90 classification, heme and hemo’s specificity is less 80% in PSSM and Hybrid, but it slightly better than oxy-50 average data. In all approaches, Ery class sensitivity data improved in oxy-90 than oxy-50 (Additional file 9: Figure S8). In PSSM method, prediction accuracy was increased than AC and DC methods.

In order to have comparison with our new and existing method (oxypred) using blind data contains 502 oxy-proteins, which were not present in our datasets. According to oxypred AC and DC methods identified 96.61% (485) and 97.81% (491) respectively. But oxypred-2 of oxy-50 models identified as 98, 99, 99, and 99% and oxy-90 models recognized as 99.20, 100, 100 and 100% in AC, DC, PSSM and Hybrid methods respectively.

### Discussion

Here, we presented an improved version of Oxypred for identifying oxy-proteins using various features [44, 45]. Here we applied two different similarity cutoff datasets. All methods recognize 100% positive and negative sequences. Hemocyanin, Hemerythrin, and Leghemoglobin classes recognizing 100% in all approaches. Oxy-50 models recognizing individual sequences as 89, 98.9 and 87.5%, and in oxy-90 models as 98, 99.8 and 98.4% identified positively as erythrocruorin, hemoglobin, and myoglobin respectively.

Further, compared with existing methods, performance based on the newly retrieved dataset, which shows nearly 97% recognition. However, our newly developed models were able to identify almost 99.99% and 100% in the oxy-50 and 90 models respectively. According to our prediction results, oxy-90 models are making a better prediction than oxy-50. However, PSSM based approaches are showing better performance in identifying oxy-proteins in both cases. Also, we found less error rate, according to confusion matrix analysis. The present oxypred2 method is able to achieve better prediction in comparison to previous method in identifying oxy-proteins. This study is an alternative method for identifying oxy-proteins and hope it will be useful to the scientific community.

## Limitations

- The exponential growth and availability of fresh annotated protein sequences in databases motivated us to develop an improved version.
- Two different sequence similarities cutoff 90 and 50% were used with various features for predicting oxy-proteins.
- The oxy-90 models are making a better prediction than oxy-50 models, and our approaches are faster and achieve a better prediction performance over the existing method.
- Finally, a web-server Oxypred2 has been developed for identifying oxygen-binding proteins.

## Additional files

**Additional file 1: Figure S1.** Amino acid distribution chart of oxy-proteins along with non-oxy, difference between 50 and 90 data.

**Additional file 2: Figure S2.** Amino acid distribution chart of oxy-proteins sub-classes (Ery, Hcy, Heme, Hemo, Leg and Myo), difference between oxy-50 and oxy-90.

**Additional file 3: Figure S3.** Sequence length profile oxy-classes. Sequence length range in histogram based on oxy-subclass organizations. X-axis for sequence length range and Y-axis for number of sequences.

**Additional file 4: Table S1.** Performance of the developed various SVM modules of oxy-proteins; amino acids (AC), dipeptides (DC), PSSM and Hybrid (AC-DC) profiles. AC- Amino acid composition, DC-dipeptide composition, PSSM position specific scoring matrix, MM- Max to Min profile. AC-DC - Hybrid is a combination of AC and DC profile. ACC-accuracy, Sen- Sensitivity, Sep-specificity, MCC- Matthews correlation coefficient. **Table S2.** Performance of various SVM modules by ROC analysis. The area under curve (AUC) for different approach for the classification of oxy-proteins. **Table S3.** Confusion Matrix. Oxypred2 developed best models performance by confusion matrix, cross checked the original oxy-class sequences, predicted by own and other models.

**Additional file 5: Figure S4.** ROC curve oxy-non-oxy in all approaches. The performance of oxypred2 models by receiver operating characteristic (ROC) plots in all approaches. The area under curve (AUC) was measured for all developed models. It is mainly to show the relationship between sensitivity and 1-specificity for each thresholds of the real value out-puts.

**Additional file 6: Figure S5.** Prediction performance of oxy-50 models. Prediction performance of the developed models on oxy-class of protein sequences. A-1, A-2, A-3, A-4, A-5 and A-6 of Ery, Hcy, Heme, Hemo, Leg and Myo models performance in AC approach. B-1, B2, B-3, B-4, B-5 and B-6 of Ery, Hcy, Heme, Hemo, Leg and Myo models performance in DC approach. C-1, C-2, C-3, C-4, C-5 and C-6 of Ery, Hcy, Heme, Hemo, Leg and Myo models performance in PSSM approach. D-1, D-2, D-3, D-4, D-5 and D-6 of Ery, Hcy, Heme, Hemo, Leg and Myo models performance in the hybrid approach. The X-axis is indexed on oxy-class proteins (Ery, Hcy, Heme, Hemo, Leg and Myo) and the Y-axis is the SVM model prediction scores.

**Additional file 7: Figure S6.** Prediction performance of oxy-90 models. Prediction performance of the developed models on oxy-class of protein sequences. E-1, E-2, E-3, E-4, E-5 and E-6 of Ery, Hcy, Heme, Hemo, Leg and Myo models performance in AC approach. F-1, F-2, F-3, F-4, F-5 and F-6 of Ery, Hcy, Heme, Hemo, Leg and Myo models performance in DC approach. G-1, G-2, G-3, G-4, G-5 and G-6 of Ery, Hcy, Heme, Hemo, Leg and Myo models performance in PSSM approach. H-1, H-2, H-3, H-4, H-5 and H-6 of Ery, Hcy, Heme, Hemo, Leg and Myo models performance in the hybrid approach. The X-axis is indexed on oxy-class proteins (Ery, Hcy, Heme, Hemo, Leg and Myo) and the Y-axis is the SVM model prediction scores.

**Additional file 8: Figure S7.** Prediction performance of the developed models accuracy (Acc), sensitivity (Sen), and specificity (Sep), performance based on the threshold from -1.5 to 1.5.

**Additional file 9: Figure S8.** Average Acc, Sen and Sep from 1.5 to -1.5 thresholds, performance compared both oxy-50 and oxy-90 datasets.

## Abbreviations

SVM: Support Vector Machine
ACC: accuracy
AC: amino acid composition
DC: dipeptide composition
PSSM: position-specific scoring matrix
Oxy-proteins: oxygen binding protein
leg: leghemoglobin
hemo: hemoglobin
myo: myoglobin
hcy: hemocyanin
ery: erythrocruorin
AUC: area under curve

## References

[1] Wakabayashi S, Matsubara H, Webster DA (1986). Primary sequence of a dimeric bacterial haemoglobin from Vitreoscilla. Nature.

[2] Weber RE, Vinogradov SN (2001). Nonvertebrate hemoglobins: functions and molecular adaptations. Physiol Rev.

[3] French CE, Bell JML, Ward FB (2008). Diversity and distribution of hemerythrin-like proteins in prokaryotes. FEMS Microbiol Lett.

[4] Svistunenko DA (2005). Reaction of haem containing proteins and enzymes with hydroperoxides: the radical view. Biochim Biophys Acta.

[5] Decker H, Terwilliger N (2000). Cops and robbers: putative evolution of copper oxygen-binding proteins. J Exp Biol.

[6] O’Brien KM, Sidell BD (2000). The interplay among cardiac ultrastructure, metabolism and the expression of oxygen-binding proteins in Antarctic fishes. J Exp Biol.

[7] Morse MP, Meyhofer E, Otto JJ, Kuzirian AM (1986). Hemocyanin respiratory pigment in bivalve mollusks. Science.

[8] Cole RP, Sukanek PC, Wittenberg JB, Wittenberg BA (1982). Mitochondrial function in the presence of myoglobin. J Appl Physiol Respir Environ Exerc Physiol.

[9] Royer WE, Strand K, van Heel M, Hendrickson WA (2000). Structural hierarchy in erythrocruorin, the giant respiratory assemblage of annelids. Proc Natl Acad Sci USA.

[10] Elmer J, Palmer AF, Cabrales P (2012). Oxygen delivery during extreme anemia with ultra-pure earthworm hemoglobin. Life Sci.

[11] Royer WE, Hendrickson WA, Love WE (1987). Crystals of Lumbricus erythrocruorin. J Mol Biol.

[12] Devos D, Valencia A (2000). Practical limits of function prediction. Proteins.

[13] Rost B, Liu J, Nair R, Wrzeszczynski KO, Ofran Y (2003). Automatic prediction of protein function. Cell Mol Life Sci.

[14] Cai YD, Doig AJ (2004). Prediction of Saccharomyces cerevisiae protein functional class from functional domain composition. Bioinformatics.

[15] Muthukrishnan S, Garg A, Raghava GPS (2007). Oxypred: prediction and classification of oxygen-binding proteins. Genomics Proteomics Bioinform.

[16] Panwar B, Gupta S, Raghava GPS (2013). Prediction of vitamin interacting residues in a vitamin binding protein using evolutionary information. BMC Bioinform.

[17] Kumar R, Panwar B, Chauhan JS, Raghava GPS (2011). Analysis and prediction of cancerlectins using evolutionary and domain information. BMC Res Notes.

[18] Garg A, Bhasin M, Raghava GPS (2005). Support vector machine-based method for subcellular localization of human proteins using amino acid compositions, their order, and similarity search. J Biol Chem.

[19] Altschul SF, Madden TL, Schäffer AA, Zhang J, Zhang Z, Miller W, Lipman DJ (1997). Gapped BLAST and PSI-BLAST: a new generation of protein database search programs. Nucleic Acids Res.

[20] Hannenhalli SS, Russell RB (2000). Analysis and prediction of functional sub-types from protein sequence alignments. J Mol Biol.

[21] Hua S, Sun Z (2001). Support vector machine approach for protein subcellular localization prediction. Bioinformatics.

[22] UniProt C (2010). The universal protein resource (UniProt) in 2010. Nucleic Acids Res.

[23] Joachims T, Scholkopf B (1999). Making large-scale SVM learning particle. Advances in Kernal Methods: support vector learning.

[24] Altschul SF, Gish W, Miller W, Myers EW, Lipman DJ (1990). Basic local alignment search tool. J Mol Biol.

[25] Bhasin M, Raghava GP (2004). Classification of nuclear receptors based on amino acid composition and dipeptide composition. J Biol Chem.

[26] Bhasin M, Raghava GPS (2004). ESLpred: SVM-based method for subcellular localization of eukaryotic proteins using dipeptide composition and PSI-BLAST. Nucleic Acids Res.

[27] Agarwal S, Mishra NK, Singh H, Raghava GPS (2011). Identification of mannose interacting residues using local composition. PLoS ONE.

[28] Jones DT (1999). Protein secondary structure prediction based on position-specific scoring matrices. J Mol Biol.

[29] Fang C, Noguchi T, Yamana H (2014). Simplified sequence-based method for ATP-binding prediction using contextual local evolutionary conservation. Algorithms Mol Biol.

[30] Mishra NK, Chang J, Zhao PX (2014). Prediction of Membrane Transport Proteins and Their Substrate Specificities Using Primary Sequence Information. PLoS ONE.

[31] Panwar B, Raghava GPS (2012). Predicting sub-cellular localization of tRNA synthetases from their primary structures. Amino Acids.

[32] Lou W, Wang X, Chen F, Chen Y, Jiang B, Zhang H (2014). Sequence based prediction of DNA-binding proteins based on hybrid feature selection using random forest and Gaussian naive Bayes. PLoS ONE.

[33] Zou C, Gong J, Li H (2013). An improved sequence based prediction protocol for DNA-binding proteins using SVM and comprehensive feature analysis. BMC Bioinform.

[34] Gromiha MM, Yabuki Y (2008). Functional discrimination of membrane proteins using machine learning techniques. BMC Bioinform.

[35] Lin HH, Han LY, Cai CZ, Ji ZL, Chen YZ (2006). Prediction of transporter family from protein sequence by support vector machine approach. Proteins.

[36] Ou YY, Chen SA, Gromiha MM (2010). Classification of transporters using efficient radial basis function networks with position-specific scoring matrices and biochemical properties. Proteins Struct Funct Bioinform.

[37] Park K-J, Kanehisa M (2003). Prediction of protein subcellular locations by support vector machines using compositions of amino acids and amino acid pairs. Bioinformatics.

[38] Chen SA, Ou YY, Lee TY, Gromiha MM (2011). Prediction of transporter targets using efficient RBF networks with PSSM profiles and biochemical properties. Bioinformatics.

[39] Mishra NK, Kumar M, Raghava GPS (2007). Support vector machine based prediction of glutathione S-transferase proteins. Protein Pept Lett.

[40] Kumar M, Gromiha MM, Raghava GPS (2007). Identification of DNA-binding proteins using support vector machines and evolutionary profiles. BMC Bioinform.

[41] Ramana J, Gupta D (2010). FaaPred: a SVM-based prediction method for fungal adhesins and adhesin-like proteins. PLoS ONE.

[42] Muthukrishnan S, Puri M, Lefevre C (2014). Support vector machine (SVM) based multiclass prediction with basic statistical analysis of plasminogen activators. BMC Res Notes.

[43] Zhang Y, Xu J, Zheng W, Zhang C, Qiu X, Chen K, Ruan J (2014). newDNA-Prot: Prediction of DNA-binding proteins by employing support vector machine and a comprehensive sequence representation. Comput Biol Chem.

[44] Garg A, Raghava GPS (2008). ESLpred2: improved method for predicting subcellular localization of eukaryotic proteins. BMC Bioinform.

[45] Muthu Krishnan S (2016). Classify vertebrate hemoglobin proteins by incorporating the evolutionary information into the general PseAAC with the hybrid approach. J Theor Biol.

