# Supplementary figures and images for: Harnessing the evolutionary information on oxygen binding proteins through Support Vector Machines based modules
Source: BMC Res Notes. 2018 May 11;11:290. doi: 10.1186/s13104-018-3383-9 (PMC5948687; doi:10.1186/s13104-018-3383-9)

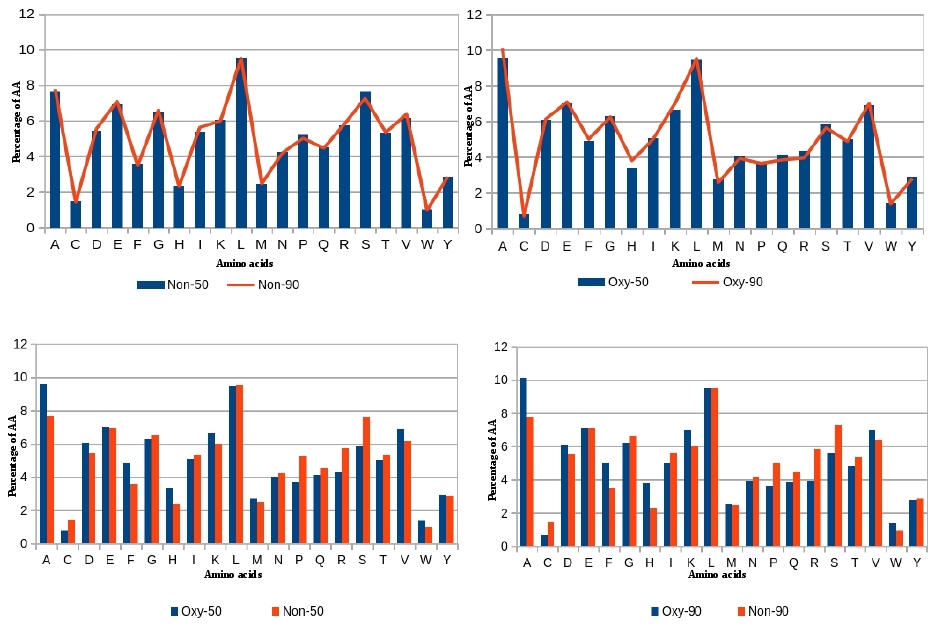

Supplement: Supplementary file 1 — Additional file 1: Figure S1. Amino acid distribution chart of oxy-proteins along with non-oxy, difference between 50 and 90 data. [file 13104_2018_3383_MOESM1_ESM.jpeg]

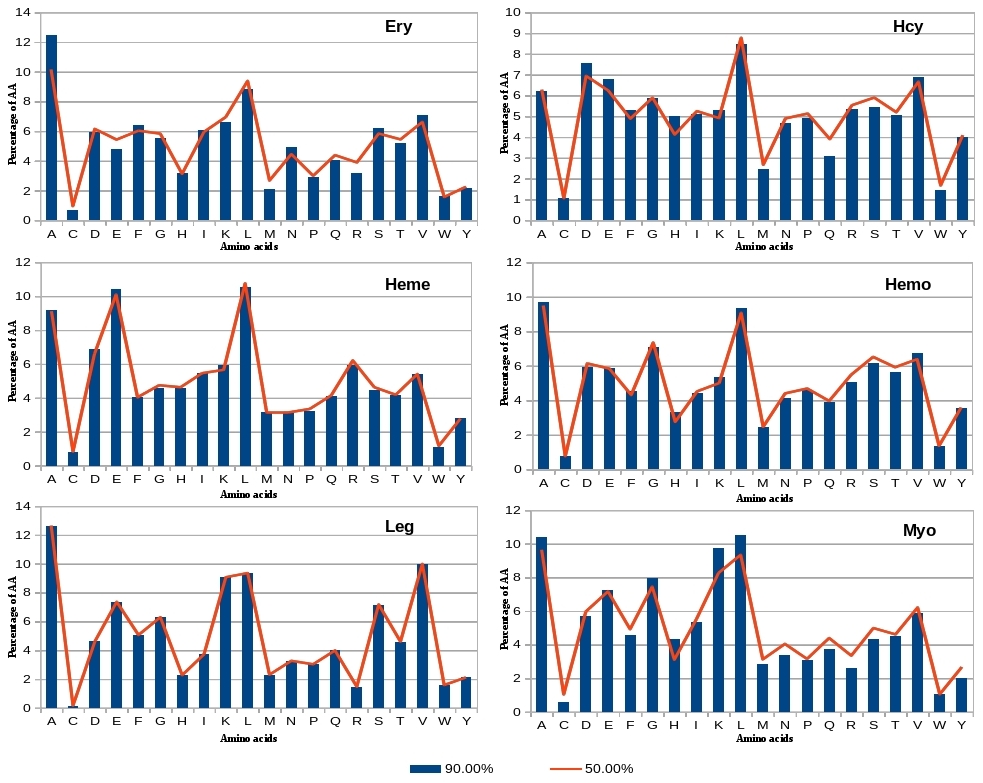

Supplement: Supplementary file 2 — Additional file 2: Figure S2. Amino acid distribution chart of oxy-proteins sub-classes (Ery, Hcy, Heme, Hemo, Leg and Myo), difference between oxy-50 and oxy-90. [file 13104_2018_3383_MOESM2_ESM.jpeg]

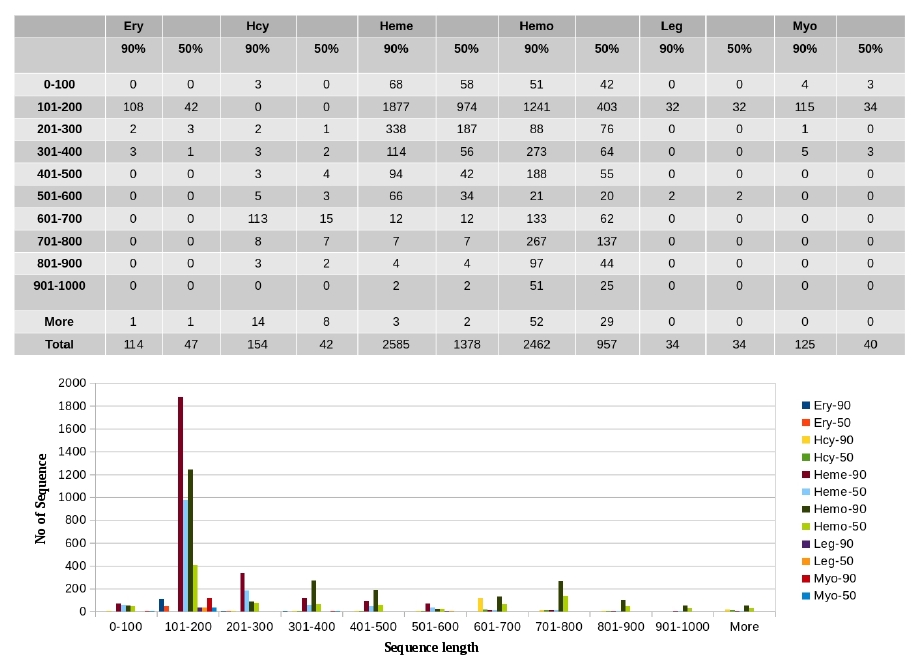

Supplement: Supplementary file 3 — Additional file 3: Figure S3. Sequence length profile oxy-classes. Sequence length range in histogram based on oxy-subclass organizations. X-axis for sequence length range and Y-axis for number of sequences. [file 13104_2018_3383_MOESM3_ESM.jpeg]

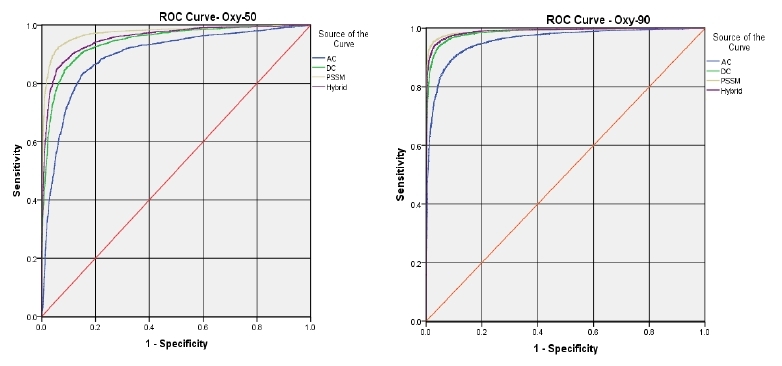

Supplement: Supplementary file 5 — Additional file 5: Figure S4. ROC curve oxy-non-oxy in all approaches. The performance of oxypred2 models by receiver operating characteristic (ROC) plots in all approaches. The area under curve (AUC) was measured for all developed models. It is mainly to show the relationship between sensitivity and 1-specificity for each thresholds of the real value out-puts. [file 13104_2018_3383_MOESM5_ESM.jpeg]

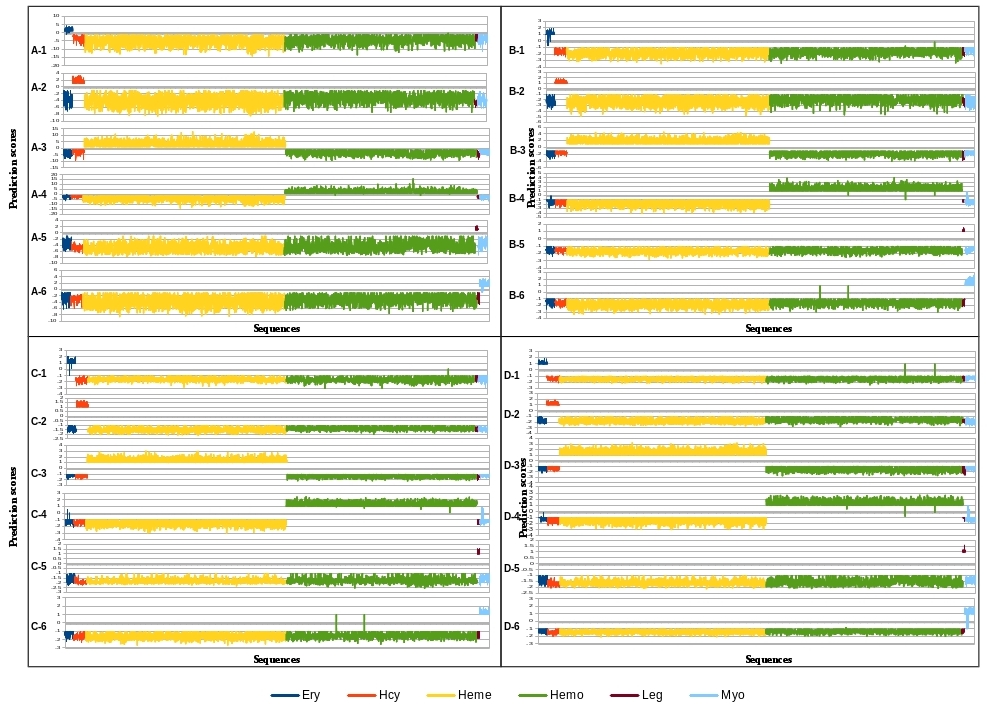

Supplement: Supplementary file 6 — Additional file 6: Figure S5. Prediction performance of oxy-50 models. Prediction performance of the developed models on oxy-class of protein sequences. A-1, A-2, A-3, A-4, A-5 and A-6 of Ery, Hcy, Heme, Hemo, Leg and Myo models performance in AC approach. B-1, B2, B-3, B-4, B-5 and B-6 of Ery, Hcy, Heme, Hemo, Leg and Myo models performance in DC approach. C-1, C-2, C-3, C-4, C-5 and C-6 of Ery, Hcy, Heme, Hemo, Leg and Myo models performance in PSSM approach. D-1, D-2, D-3, D-4, D-5 and D-6 of Ery, Hcy, Heme, Hemo, Leg and Myo models performance in the hybrid approach. The X-axis is indexed on oxy-class proteins (Ery, Hcy, Heme, Hemo, Leg and Myo) and the Y-axis is the SVM model prediction scores. [file 13104_2018_3383_MOESM6_ESM.jpeg]

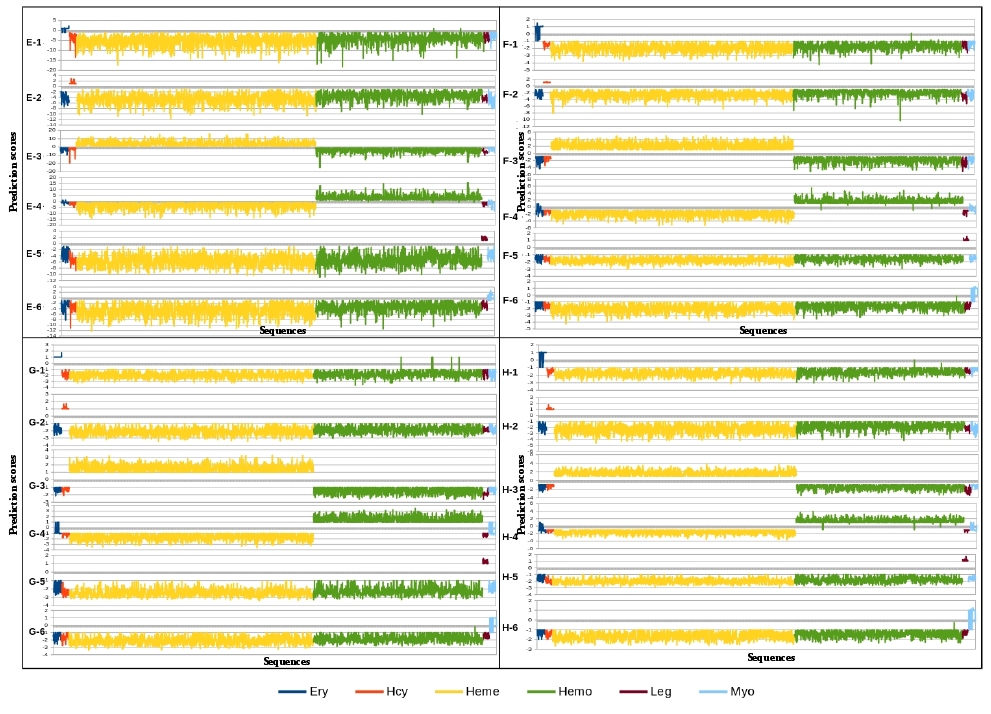

Supplement: Supplementary file 7 — Additional file 7: Figure S6. Prediction performance of oxy-90 models. Prediction performance of the developed models on oxy-class of protein sequences. E-1, E-2, E-3, E-4, E-5 and E-6 of Ery, Hcy, Heme, Hemo, Leg and Myo models performance in AC approach. F-1, F-2, F-3, F-4, F-5 and F-6 of Ery, Hcy, Heme, Hemo, Leg and Myo models performance in DC approach. G-1, G-2, G-3, G-4, G-5 and G-6 of Ery, Hcy, Heme, Hemo, Leg and Myo models performance in PSSM approach. H-1, H-2, H-3, H-4, H-5 and H-6 of Ery, Hcy, Heme, Hemo, Leg and Myo models performance in the hybrid approach. The X-axis is indexed on oxy-class proteins (Ery, Hcy, Heme, Hemo, Leg and Myo) and the Y-axis is the SVM model prediction scores. [file 13104_2018_3383_MOESM7_ESM.jpeg]

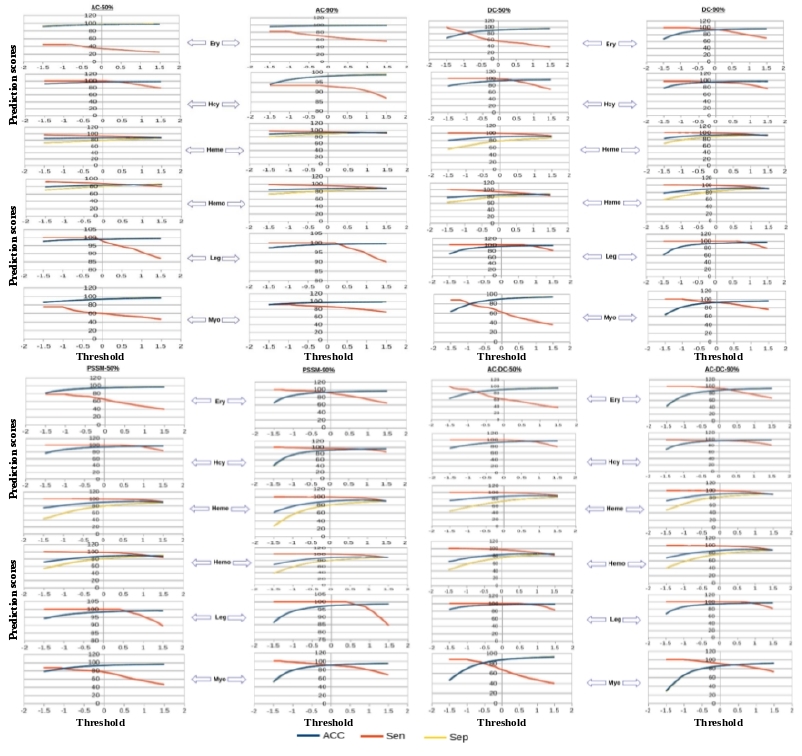

Supplement: Supplementary file 8 — Additional file 8: Figure S7. Prediction performance of the developed models accuracy (Acc), sensitivity (Sen), and specificity (Sep), performance based on the threshold from -1.5 to 1.5. [file 13104_2018_3383_MOESM8_ESM.jpeg]

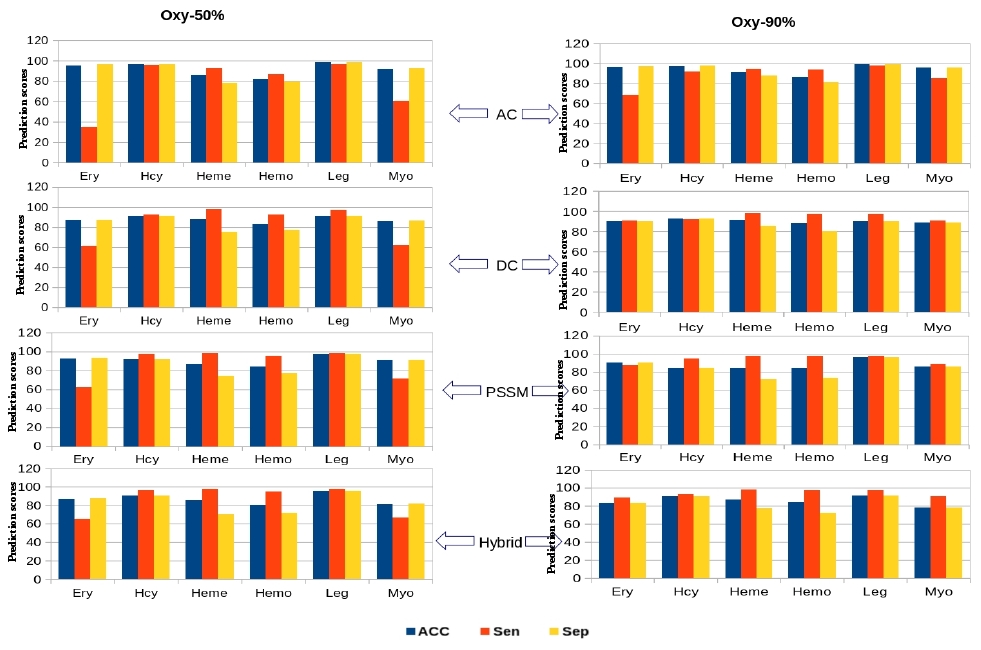

Supplement: Supplementary file 9 — Additional file 9: Figure S8. Average Acc, Sen and Sep from 1.5 to -1.5 thresholds, performance compared both oxy-50 and oxy-90 datasets. [file 13104_2018_3383_MOESM9_ESM.jpeg]
