# Supplementary material for: Harnessing the evolutionary information on oxygen binding proteins through Support Vector Machines based modules
Source: BMC Res Notes. 2018 May 11;11:290. doi: 10.1186/s13104-018-3383-9 (PMC5948687; doi:10.1186/s13104-018-3383-9)
Supplement: Supplementary file 4 — Additional file 4: Table S1. Performance of the developed various SVM modules of oxy-proteins; amino acids (AC), dipeptides (DC), PSSM and Hybrid (AC-DC) profiles. AC- Amino acid composition, DC-dipeptide composition, PSSM position specific scoring matrix, MM- Max to Min profile. AC-DC - Hybrid is a combination of AC and DC profile. ACC-accuracy, Sen- Sensitivity, Sep-specificity, MCC- Matthews correlation coefficient. Table S2. Performance of various SVM modules by ROC analysis. The area under curve (AUC) for different approach for the classification of oxy-proteins. Table S3. Confusion Matrix. Oxypred2 developed best models performance by confusion matrix, cross checked the original oxy-class sequences, predicted by own and other models. [file 13104_2018_3383_MOESM4_ESM.doc]

**Additional Table S1 :** Performance of the developed various SVM modules of oxy-proteins; amino acids (AC), dipeptides (DC), PSSM and Hybrid (AC-DC) profiles.

**
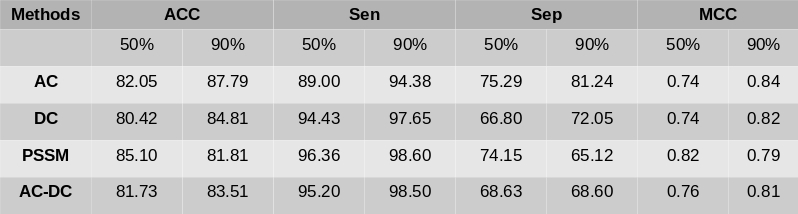
**

AC- Amino acid composition, DC-dipeptide composition, PSSM position specific scoring matrix, MM- Max to Min profile. AC-DC - Hybrid is a combination of AC and DC profile. ACC-accuracy, Sen- Sensitivity, Sep-specificity, MCC- Matthews correlation coefficient

**Additional Table S2:** Performance of various SVM modules by ROC analysis. The area under curve (AUC) for different approach for the classification of oxy-proteins.

**
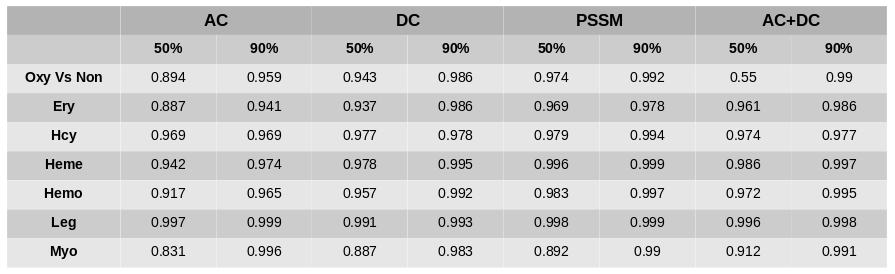
**

**Additional Table S3 : Confusion Matrix.**  Oxypred2 developed best models performance by confusion matrix, cross checked the original oxy-class sequences, predicted by own and other models.

|  |  | **Total** | | **Ery** | | **Hcy** | | **Heme** | | **Hemo** | | **Leg** | | **Myo** | | **NOT** | |
| --- | --- | --- | --- | --- | --- | --- | --- | --- | --- | --- | --- | --- | --- | --- | --- | --- | --- |
|  |  | **50%** | **90%** | **50%** | **90%** | **50%** | **90%** | **50%** | **90%** | **50%** | **90%** | **50%** | **90%** | **50%** | **90%** | **50%** | **90%** |
| **Ery** | **AC** | **47** | **114** | **42** | **112** |  |  |  |  | **2** |  |  |  |  |  | **3** | **2** |
|  | **DC** |  |  | **42** | **112** |  |  |  |  | **1** |  |  |  |  |  | **4** | **2** |
|  | **PSSM** |  |  | **42** | **112** |  |  |  |  | **5** | **1** |  |  |  |  |  | **1** |
|  | **AC-DC** |  |  | **42** | **112** |  |  |  |  | **1** | **2** |  |  |  |  | **4** |  |
| **Hcy** | **AC** | **43** | **154** |  |  | **43** | **154** |  |  |  |  |  |  |  |  |  |  |
|  | **DC** |  |  |  |  | **43** | **154** |  |  |  |  |  |  |  |  |  |  |
|  | **PSSM** |  |  |  |  | **43** | **154** |  |  |  |  |  |  |  |  |  |  |
|  | **AC-DC** |  |  |  |  | **43** | **154** |  |  |  |  |  |  |  |  |  |  |
| **Heme** | **AC** | **1378** | **2584** |  |  |  |  | **1378** | **2584** |  |  |  |  |  |  |  |  |
|  | **DC** |  |  |  |  |  |  | **1378** | **2584** |  |  |  |  |  |  |  |  |
|  | **PSSM** |  |  |  |  |  |  | **1378** | **2584** |  |  |  |  |  |  |  |  |
|  | **AC-DC** |  |  |  |  |  |  | **1378** | **2584** |  |  |  |  |  |  |  |  |
| **Hemo** | **AC** | **957** | **2462** | **2** | **1** |  |  |  |  | **947** | **2458** |  |  | **3** | **1** | **5** | **2** |
|  | **DC** |  |  | **3** |  |  |  |  |  | **947** | **2458** |  |  | **4** | **2** | **3** | **2** |
|  | **PSSM** |  |  | **5** | **2** |  |  |  |  | **947** | **2458** |  |  | **5** | **2** |  |  |
|  | **AC-DC** |  |  | **3** |  |  |  |  |  | **947** | **2458** |  |  | **4** | **2** | **3** | **2** |
| **Leg** | **AC** | **34** | **34** |  |  |  |  |  |  |  |  | **34** | **34** |  |  |  |  |
|  | **DC** |  |  |  |  |  |  |  |  |  |  | **34** | **34** |  |  |  |  |
|  | **PSSM** |  |  |  |  |  |  |  |  |  |  | **34** | **34** |  |  |  |  |
|  | **AC-DC** |  |  |  |  |  |  |  |  |  |  | **34** | **34** |  |  |  |  |
| **Myo** | **AC** | **40** | **125** |  |  |  |  |  |  |  |  |  |  | **35** | **123** | **5** | **2** |
|  | **DC** |  |  |  |  |  |  |  |  |  | **2** |  |  | **35** | **123** | **5** |  |
|  | **PSSM** |  |  |  |  |  |  |  |  |  | **2** |  |  | **35** | **123** | **5** |  |
|  | **AC-DC** |  |  |  |  |  |  |  |  |  |  |  |  | **35** | **123** | **5** | **2** |
